# National Medical Convention

**Published:** 1846-06

**Authors:** 


### NATIONAL MEDICAL CONVENTION.

*New York, May* 5, 1846.

At a meeting of the National Medical Convention, pursuant to the call of the New York State Medical Society, convened in the building of the Medical Department of the University of the city of New York, on motion of Dr. Edward Delafield, of New York, Dr. Bell, of Philadelphia, was appointed Chairman, and Dr. Buel, of New York, Secretary, to act until the Convention be duly organised.

Drs. Baxley, of Maryland, Davis, of New York, and Arnold, of Georgia, were appointed a committee to receive the credentials of Delegates.

The Committee, after having performed that duty, made the following report:—

The Committee appointed to examine the credentials of the Delegates to the National Medical Convention, recommended to be held by the New York State Medical Society, in the city of New York, report, that they have performed that duty.

The Committee have felt themselves embarrassed by conflicting resolutions passed by the New York Society in 1845 and 1846, viz.: in 1845, it was "*Resolved*, that the New York State Medical Society earnestly recommend a *National Convention of Delegates* from Medical Societies and Colleges in the whole *Union*, to convene in the city of New York, on the first Tuesday in May, in the year 1846, for the purpose of adopting some *concerted action* on the subject set forth in the foregoing preamble."

In February, 1846, it was "*Resolved*, That the preamble and resolutions passed by this Society at its annual session, Feb. 6, 1845, did not contemplate the appointment of Delegates to the National Convention, by County or merely local Societies in those States where Delegates *are* appointed by a regularly organised *State* Society."

They have therefore thought proper to report the names of all gentlemen from Medical Societies, Colleges, and Institutions, of all the States who come properly accredited, in accordance with the original resolution, and leave it to the Convention to determine whether such shall constitute the National Medical Convention; or whether the interpretation of the resolution of 1845, adopted by the N. Y. Society in 1846, which excludes delegates from local or voluntary societies from States sending delegates from regularly organised State Societies shall be strictly adhered to.

Respectfully submitted,
H. W. Baxley,
N. S. Davis,
Richard D. Arnold,

The following list of Delegates was then handed in:—

FROM VERMONT.

*Society of the Alumni of Castleton Medical College.*—Drs. Simeon A. Cook, Joseph Perkins, Egbert Jamieson, Horace Green.

*Vermont Medical College.*—Dr. Alonzo Clark.

FROM NEW HAMPSHIRE.

*Centre District N. H. Medical Society.*—Drs. Charles P. Gage, Richard P. J. Tenney.

FROM MASSACHUSETTS.

*Berkshire Medical Institute.*—Dr. Alonzo Clark.

FROM CONNECTICUT.

*State Medical Society.*—Drs. V. M. Dow, Rufus Blakewood, William H. Cogswell, J. G. Beckwith, Eleazar Hunt, D. T. Brainard, Richard Warner.

*Medical Institution of Yale College.*—Drs. J. Ives, J. Knight.

FROM NEW YORK.

*Medical Society of City and County of New York.*—Drs. James R. Manley, Isaac Wood, John W. Francis, Benjamin Drake, Gilbert Smith, W. W. Minor, F. U. Johnston, James Stewart, Thomas Chalmers, H. D. Bulkley, W. P. Buel, J. R. Wood, John S. Heard, A. N. Gunn, B. R. Robson, John Watson, J. S. Ferguson, R. L. Morris, R. T. Underhill, S. P. White, J. R. Van Cleek, J. C. Cheesman, A. C. Post, G. Buck, O. S. Bartles.

*N. Y. Medical and Surgical Society.*—Drs. F. Campbell Stewart, J. A. Swett, Edward L. Beadle.

*Bloomingdale Asylum*—Dr. Pliny Earle.

*Kings Co. Medical Society.*—Drs. Chauncey L. Mitchell, Bradley Parker, J. Sullivan Thorne, T. L. Mason.

*Alumni Geneva Medical College.*—Drs. Peter Wilson, H. M. Gray.

*Medical Faculty of College of Physicians and Surgeons.*—Drs. John B. Beck, Willard Parker.

*Medical Society of State of New York.*—Drs. John Stearns, Stephen Hasbrouck, Merrit H. Cash, S. M. Crawford, Joel A. Wing, Daniel Ayres, Darius Clark, Thos. W. Blatchford, Sumner Ely, John McCall, N. S. Davis, Augustus Willard, L. G. Tefft, Alexander McIntyre, Maltby Strong, Charles Winnie.

*Madison Co. Medical Society.*—Dr. D. E. Hurd.

*New York Hospital.*—Dr. J. H. Griscom.

*University of the City of New York.*—Drs. G. S. Pattison, G. S. Bedford.

*Buffalo Medical Association.*—Dr. Bryant Burwell.

*Erie Co. Medical Society.*—Dr. Austin Flint.

*Albany Medical College.*—Drs. J. McNaughton, A. March.

*Genesee Medical Society.*—Dr. John Coates.

*Trustees of the College of Physicians and Surgeons.*—Drs. A. H. Stevens, Thomas Cock, Edward Delafield.

*Geneva Medical College.*—Dr. Charles A. Lee.

FROM PENNSYLVANIA.

*Philadelphia Medical Soeiety.*—Drs. John Bell, Henry Bond, Geo. W. Norris, Isaac Hays, Isaac Parrish, Joseph Warrington, Alfred Stillé, J. Rodman Paul, Francis West, Gouverneur Emerson, Caspar Morris, Meredith Clymer.

*Medical Department of Pennsylvania College.*—Drs. H. S. Patterson, W. A. Atlee.

FROM DELAWARE.

*Medical Association of Wilmington.*—Dr. Lewis P. Bush.

*Medical Society of Delaware.*—Drs. James W. Thompson, E. S. Richard, William W. Stewart, William Cummings, Gove Saulsbury, James Couper.

FROM MARYLAND.

*Washington Medical College.*—Drs. H. Baxley, Charles Bell Gibson.

FROM VIRGINIA.

*Medical Society of Virginia.*—Drs. Robert W. Haxall, Samuel A. Patteson, Charles Mills, Frederick Marx, James Conway, J. Cullen.*

FROM GEORGIA.

*Georgia Medical Society.*—Dr. Richard D. Arnold.

FROM MISSISSIPPI.

*Mississippi State Medical Society.*—Drs. E. D. Fenner, of New Orleans, C. S. Magoun.

FROM INDIANA.

*La Porte University.*—Dr. Azariah B. Shipman.

FROM ILLINOIS.

*Medical Department of Illinois College.*—Drs. Edward Mead, William A. Cheatham.

FROM TENNESSEE.

*Medical Society of Tennessee.*—Dr. Wm. A. Cheatham.

It was on motion *Resolved*, that the Committee be continued to receive the credentials of such Delegates as may hereafter arrive.

On motion of Dr. Arnold, it was *Resolved*, that all gentlemen who have presented credentials from any regularly organised Medical Society in this Union, be considered members of this Convention.

It was moved by Dr. Davis, and carried, that Dr. Theophilus C. Dunn, President of the Rhode Island State Medical Society, be invited to take a seat as a member of this Convention.

It was moved by Dr. Underhill, and duly seconded, that all medical gentlemen in good standing, who may be present from States not otherwise represented, be admitted as delegates, which motion was carried.

Drs. E. C. Marsh, and Lyndon A. Smith, of New Jersey, were invited to take seats under the above resolution.

On motion, a Committee of one from each State represented in the Convention, was appointed to nominate officers for the Convention.

The following gentlemen were appointed:

From New Hampshire, Dr. Gage; Vermont, Dr. Cook; Massachusetts, Dr. Clark; Rhode Island, Dr. Dunn; Connecticut, Dr. Knight; New York, Stearns; New Jersey, Marsh; Pennsylvania, Bond; Delaware, Bush; Maryland, Baxley; Virginia, Haxall; Georgia, Arnold; Mississippi, Fener; Indiana, Shipman; Illinois, Mead; Tennessee, Cheatham.

*Dr. Cullen represented the Hampden Sydney College, (Medical Department,) Richmond.—*Ed. Bulletin.*

The Committee, after having retired, returned and made the following report:

The Committee appointed to nominate suitable officers to preside over the Convention, report that they have unanimously agreed to propose the names of the following gentlemen:—

*For President.*—Dr. J. Knight, of New Haven.

*For Vice Presidents.*—Dr. John Bell, of Philadelphia; Dr. Edward Delafield, of New York City.

*For Secretaries.*—Dr. Richard D. Arnold, of Savannah; Dr. Alfred Stillé, of Philadelphia.

The Report was adopted:—*Committee discharged.*

The Officers, with the exception of Dr. Delafield, took their seats.

Dr. Bedford, of the University of the city of New York, moved the following preamble and resolution, seconded by Dr. Pattison, also of the University of the city of New York:—

Whereas, the call of the State Medical Society of New York, for a National Medical Convention to be held in the city of New York on the first Tuesday in May, has failed in a representation from one-half the United States, and from a majority of the Medical Colleges; and whereas the State Medical Society has emphatically stated that there is no mode of accomplishing the object of the Convention, without concert of action on the part of the Medical Societies, Colleges and Institutions of *all* the States, therefore, Resolved, that this Convention adjourn *sine die.*

The Yeas and Nays were called on this, and were as follows:—

*Yeas*—Drs. Bedford and Pattison—2.

*Nays*—Drs. S. A. Cook, H. Green, C. P. Gage, R. P. J. Tenney, A. Clark, W. H. Cogswell, J. G. Beckwith, R. Warner, D. T. Brainard, B. Burwell, A. Flint, J. McNaughton, A. March, J. Coates, F. C. Stewart, J. A. Swett, E. L. Beadle, C. A. Lee, J. Bell, H. Bond, G. W. Norris, I. Hays, I. Parrish, J. L. Warrington, A. Stillé, J. R. Paul, F. West, G. Emerson, M. Clymer, W. A. Atlee, L. P. Bush, E. S. Richards, G. Saulsbury, J. Couper, H. W. Baxley, R. W. Haxall, S. A. Patteson, J. Cullen, R. D. Arnold, E. D. Fenner, A. B. Shipman, E. Mead, W. A. Cheatham, P. Wilson, D. E. Hurd, S. Wood, G. Smith, H. D. Bulkley, W. P. Buel, J. K. Wood, J. S. Heard, A. N. Gunn, J. Watson, R. L. Morris, R. T. Underhill, S. P. White, J. C. Cheeseman, A. C. Post, G. Buck, O. S. Bartles, S. Hasbrouck, J. A. Wing, D. Ayres, N. S. Davis, A. Willard, P. Earle, T. Cock, C. L. Mitchell, J. S. Thorne, T. S. Dunn, E. J. Marsh, L. A. Smith, W. W. Stuart, and J. Knight—74.

Dr. Clymer then offered the following resolution:—

*Resolved*, That a Committee be appointed to provide other accommodations for the sittings of this Convention, than in the University of New York.

Several amendments were offered to this resolution, but while they were pending, Dr. Haxall moved to lay the whole upon the table, which was carried—Yeas 34, Nays 31.

Dr. N. S. Davis moved the following, which was adopted:—

*Resolved*, That a Committee of nine be appointed to bring the subject of Medical Education before the Convention in the form of distinct propositions, suitable for discussion and action, and that it report at the next meeting.

The following gentlemen were appointed:

Drs. N. S. Davis, March, Hays, Watson, Brainard, Stearns, Bush, Haxall, Bell.

On motion of Dr. R. Wood, of New York, the President was added to the Committee.

Dr. Buel offered the following, which was adopted:

*Resolved*, That the Committee be instructed to receive and submit propositions on all subjects proper to be brought before this Convention.

On motion the Convention adjourned until 10 o'clock next morning.

*Wednesday, May 6th.*

The Convention met pursuant to adjournment. The roll was called, and 63 delegates answered to their names. The minutes of the last meeting were then read and confirmed. The Chairman of the Committee on Credentials announced the presence of Dr. C. A. Pope, delegate from the Medical Society of the State of Missouri, and of Drs. Anderson, G. Dana, J. Spaulding, and J. A. Allen; delegates from the Vermont Medical Society.

Dr. N. S. Davis, Chairman of the Committee, appointed at the last meeting to present the subject of Medical Education to the Convention in a form proper for discussion, and, generally, to prepare business for its action, reported that, owing to the short time allowed them, the Committee had not been able to agree in regard to the subject especially entrusted to them, but that they had unanimously determined to lay before the Convention certain resolutions which they believe adapted to fulfil the immediate objects contemplated by the Convention. Whereupon Dr. Hays, from the same Committee, submitted the following preamble and resolutions:

Whereas, it has been shown by experience, that the Association of persons engaged in the same pursuit, facilitates the attainment of their common objects, therefore—

1st. *Resolved*, that it is expedient for the medical profession of the United States, to institute a *National Medical Association*, for the protection of their interests, for the maintainance of their honour and respectability, for the advancement of their knowledge, and for the extension of their usefulness.

2d. *Resolved*, that a Committee of seven be appointed to report a plan of organisation for such an Association, at the meeting to be held in Philadelphia, on the first Wednesday in May, 1847.

3d. *Resolved*, that a Committee of seven be appointed to prepare and issue an address to the different regularly organised Medical Societies and chartered Medical Schools in the United States, setting forth the objects of the National Medical Association, and inviting them to send delegates to a Convention, to be held in Philadelphia, on the first Wednesday in May, 1847.

4th, *Resolved*, that it is desirable that a uniform and elevated standard of requirements for the degree of M. D., should be adopted by all the Medical Schools in the United States, and that a Committee of seven be appointed to report on this subject at the meeting to be held in Philadelphia, on the first Wednesday in May, 1847.

5th. *Resolved*, that it is desirable that young men, before being received as students of medicine, should have acquired a suitable preliminary education, and that a Committee of seven be appointed to report on the standard of acquirements which should be exacted of such young men, and to report at the meeting to be held on the first Wednesday in May, 1847.

6th. *Resolved*, that it is expedient that the medical profession of the United States should be governed by the same code of Medical Ethics, and that a Committee of seven be appointed to report a code for that purpose, at the meeting to be held at Philadelphia, on the first Wednesday in May, 1847.

Dr. J. S. Copes was announced to be a duly authorised delegate from the Medical Society of Mississippi; and Dr. G. H. White, of the Hudson Lunatic Asylum was admitted to a seat in the Convention.

Dr. G. S. Patterson moved that the Report of the Committee on Business be adopted. Carried, *nem. diss.*

Dr. Stearns moved that the resolutions be considered *seriatim.* Carried.

The preamble and 1st resolution were then adopted *nem. diss.*

The 2d resolution being under discussion, Dr. S. Hasbrouck moved that the committee to be appointed under that resolution, report on tomorrow morning. After some remarks by Drs. Haxall, Thomson, and Davis, the motion was withdrawn.

Dr. Griscom moved that the next Convention be held in September, 1847. After some remarks by delegates from the South, declaring that physicians of that region could not possibly be spared from their duties in the autumn, the motion was rejected.

The 2d resolution and then the 3d, were adopted without further discussion, *nem. diss.*

Dr. F. Hasbrouck, as Superintendent of the N. Y. City Lunatic Asylum, was here admitted to a seat in the Convention.

The 4th resolution was then adopted, *nem. diss.*

The 5th resolution, after some observations from Drs. Manley and H. S. Patterson, and the loss of an amendment proposed by the latter gentlemen, was adopted, *nem. diss.*

The 6th resolution was in like manner adopted, without debate; after which, on motion of Dr. Bush, the preamble and resolutions collectively were unanimously adopted.

The Convention then took a recess for half an hour, after empowering the President to fill the Committees called for by the resolutions just passed.

On reassembling, the Convention received Dr. G. Sumner, a delegate from the Medical Society of Connecticut.

Dr. Clymer asked and received permission to correct an error personal to himself in a report of a morning newspaper (the N. Y. Herald,) concerning the vote on Dr. Haxall's motion to lay on the table the resolution offered by Dr. Clymer for adjourning forthwith from the rooms of the Faculty of the University of New York city. The newspaper in question had stated that the only serious opposition to laying this resolution on the table proceeded from the delegates of the College of Physicians and Surgeons, whereas the vote had really stood 34 in favour, and 31 against disposing of the resolution in this manner.

Dr. J. B. Beck, of the Faculty of the College of Physicians and Surgeons, stated that so far from any opposition to laying the resolution on the table having proceeded from the body which he represented, neither he nor his colleague, Dr. Parker, the only delegates from that body, were even so much as present during the discussion, or at the vote, on the resolution.

The Secretary asked permission to insert the correction in the minutes of the last meeting as confirmed; stating that the vote was upon the original record of the proceedings, but had been omitted in transcription. Leave was granted.

Dr. Sumner moved to reconsider the 2d resolution; the motion, after remarks by Drs. Baxley, Haxall, and Davis, was laid on the table.

Dr. Bartles introduced the following resolution:

*Resolved*, That the union of the business of *Teaching* and *Licensing* in the same hands is wrong in principle, and liable to great abuse in practice. Instead of conferring the right to license on Medical Colleges, and State and County Medical Societies, it should be restricted to one Board in each State, composed in fair proportion of representatives from its Medical Colleges and the profession at large, and the pay for whose services as Examiners should in no degree depend on the number licensed by them.

Dr. Sumner moved to lay the resolution on the table. After remarks by Drs. F. C. Stewart and Clymer, the motion was withdrawn.

Dr. Parrish moved that the resolution be referred to the Committee under the 4th resolution reported by the Committee on Business.

Dr. Baxley moved an amendment, which was accepted by Dr. Parrish, providing that all communication upon the subject of medical degrees be referred to the same Committee.

Dr. Manley remarked at length upon the subject of Medical Education. Dr. Baxley replied. Dr. Manley rejoined; and then moved to refer the resolution introduced by Dr. Bartles to a special Committee.

On motion, the subject was temporarily laid upon the table in order that the President might announce the Committees he had appointed.

They were as follows:

*Under the 2d Resolution.*—Drs. J. Watson, J. Stearns, F. C. Stewart, N. Y.; A. Stillé, Philadelphia; N. S. Davis, Binghampton, N. Y.; Cogswell, N. Haven, Conn.; Fenner, New Orleans.

*Uuder the 3d Resolution.*—Drs. E. Ives, Dow, and Sumner, of N.

Haven, Conn.; J. McNaughton, Albany, N. Y., Blatchford, Troy, N. Y.; Burwell, Buffalo, N. Y.; and Baxley, Baltimore, Md.

*Under the 4th Resolution.*—Drs. Haxall, and Cullen, Richmond, Va.; S. A. Patteson, Manchester, Va.; G. W. Norris, Philadelphia; A. Flint, Buffalo, N. Y.; J. Perkins, Castleton, Vt.; and J. A. Wing, Albany, N. Y.

*Under the 5th Resolution.*—Drs. J. Couper, Newcastle, Del.; L. P. Bush, and J. W. Thompson, Wilmington, Del.; E. Mead, Jacksonville, Ill.; A. March, Albany, N. Y.; J. Atlee, Philadelphia; and D. J. Brainard, New London, Conn.

*Under the 6th Resolution.*—Drs. Bell, Hays, and Emerson, Philadelphia, Morris, Dover, Del.; J. C. Dunn, Newport, R. I.; Alonzo Clark, N. Y.; and R. D. Arnold, Savannah, Ga.

Dr. Haxall moved that the President be added, as Chairman to the Committee under the 3d resolution. Carried unanimously.

The consideration of Dr. Bartles' resolution, as amended by Dr. Manley, was then resumed, and it was ordered that when the vote upon it is taken, it be taken by yeas and nays.

Drs. S. Hasbrouck and Davis remarked at length upon the subject of the resolution. Observations were also made by Drs. Baxley, and Underhill.

Dr. Sumner moved to lay the Resolution on the table. Lost by a vote of 34 to 40.

Dr. Parrish having withdrawn his amendment, the yeas and nays on Dr. Bartle's resolution, as amended by Dr. Manley, were called, when it was adopted by the following vote:

*Yeas.*—Drs. Arnold, Ayres, Bartles, Beadle, Beck, Bell, Bulkley, Barwell, Bond, Buck, Buel, Cheesman, Cock, Coates, Copes, Cummins, Cook, Davis, Delafield, Drake, Fenner, Ferguson, Gage, Gray, Griscom, Gunn, F. Hasbrouck, S. Hasbrouck, Heard, McGoun, McNaughton, Mitchell, R. L. Morris, B. Parker, W. Parker, Paul, Parrish, Pope, Rickards, Saulsbury, F. C. Stewart, W. W. Stewart, L. A. Smith, Stillé, Stearns, Swett, Thorne, Underhill, Van Kleek, Warner, Watson, Warrington, West, Willard, S. P. White, G. H. White, Wing, J. Wood, J. R. Wood.—59.

*Nays.*—Drs. Baxley, Beckwith, Bedford, Blakeman, Brainard, Bush, Cheatham, A. Clark, Clymer, Couper, Cullen, Emerson, Flint, Gibson, Hays, Haxall, Lee, March, H. S. Patterson, G. S. Pattison, S. A. Pateson, Shipman, Sumner, Thompson.—24.

Dr. Griscom moved that a Committee of *five* be appointed to consider the *expediency*, and if expedient, the *mode*, of recommending and urging upon the several State governments the adoption of measures for a registration of the births, marriages, and deaths, of their several populations. Carried, *nem. diss.*

Dr. McGoun moved that the thanks of the Convention be presented to the officers of this Convention, for the prompt and efficient discharge of their duties. Carried, *nem. diss.*

Dr. Griscom, introduced the following resolution:

*Resolved,* That Mr. Lemuel Shattuck, of Boston; Drs. Jarvis, of

Dorchester, Mass.; Emerson, of Philadelphia; T. R. Beck, of Albany; and C. A. Lee, of N. Y.; be a Committee to prepare a nomenclature of diseases, adapted to the United States, having reference to a general registration of deaths; to report to a future Convention, which was adopted; and on motion, Dr. Griscom was added to the Committee as Chairman.

Dr. Swett, of N. Y., offered the following, which after receiving the approbation of the delegates from New York, was adopted:

*Resolved*, That a publication of the proceedings of this Convention be made in pamphlet form; that ten thousand copies be printed; that the New York City delegation assume the expense; and that it be referred to the first Standing Committee.

The President then announced the Committees appointed by him, under the resolutions of Dr. Bartles, and of Dr. Griscom, as follows:—

Under Dr. Bartles, in reference to the separation of teaching and licensing: Drs. McNaughton, Albany, N. Y.; J. R. Manley, N. Y.; J. W. Francis, N. Y.; Isaac Parrish, Philada.; R. Blakeman, Fairfield, Conn.; J. Cullen, Richmond, Va.; and Thomas Cock, N. Y.

Under that of Dr. Griscom, in reference to the subject of registration: Drs. J. H. Griscom, N. Y.; G. Emerson, Philada.; A. Clark, N. Y.; C. A. Lee, Geneva, N. Y.; and James Stewart, N. Y.

The following resolution, offered by Dr. Thomson, of Delaware, and seconded by Dr. Clymer, of Philadelphia, was unanimously adopted:

*Resolved*, That this Convention hereby tender to the Medical Faculty of the City of New York, and to the Trustees of the College of Physicians and Surgeons of the State of New York, its unanimous thanks for the courteous offer of their respective buildings for the sitting of this Convention.

Dr. Bell introduced the following resolution, which was adopted, *nem. diss.*

*Resolved*, That the Convention give its approval to the objects and labours of the Sydenham Society.

Dr. Cogswell offered the following resolution, which was unanimously adopted:

*Resolved*, That the thanks of this Convention be presented to the President, for the able, gentlemanly, and impartial manner in which he has discharged the duties of the Chair.

Upon motion of Dr. Hays, the Convention then adjourned, *sine die.*

